# Supplementary material for: An integrated atom array-nanophotonic chip platform with background-free imaging
Source: Nat Commun. 2024 Jul 22;15:6156. doi: 10.1038/s41467-024-50355-4 (PMC11263554; doi:10.1038/s41467-024-50355-4)
Supplement: Supplementary file 1 — Supplementary Information [file 41467_2024_50355_MOESM1_ESM.pdf]

Supplementary Information

An integrated atom array-nanophotonic chip platform with background-free imaging

1 Device Fabrication

The nanophotonic devices on the silicon nitride layer are patterned using conventional electron-beam lithography (EBL) with a ZEP 520A resist etch mask, followed by CHF_3/O_2 inductively coupled reactive-ion etching (RIE). Once the devices are patterned, the chip region of 2 mm $\times$ 8 mm around the devices is defined using photolithography with a AZ4620 resist etch mask. This is followed by deep reactive-ion etching (DRIE) and dicing to isolate the chip region. After protecting the silicon nitride device side using a double layer PMMA mask, a final KOH etching step is performed to undercut the silicon from the device region by etching from the sides. The undercut devices are shown in Fig. S1.

2 Loading Atoms onto Devices

The atoms are loaded in the "loading region" (as marked in Fig. 2**b**) and then translated along the X-direction until they are in between the devices. At this position, the Stark shift from the tweezer is similar to that of a free-space tweezer as depicted in Supplementary Fig. S2**a**. From here, atoms are loaded onto the devices by performing a Y-direction move, with a speed of 6 μm/ms. As the tweezer approaches the device, the partial reflection of the tweezer from the surface interferes with the incident beam causing a lattice to form, where each intensity maximum can trap atoms. The corresponding modified Stark shift on the atoms is shown in Fig. S2**b**. The proportion of atoms that load into various intensity maxima is optimized by adjusting the focal plane of the tweezer with respect to the device position. Previous work has shown up to 94% loading efficiency into the closest intensity maximum of the standing wave potential, and Monte Carlo simulations show that up to 100% loading efficiency can be achieved [1, 2]. For the device thickness

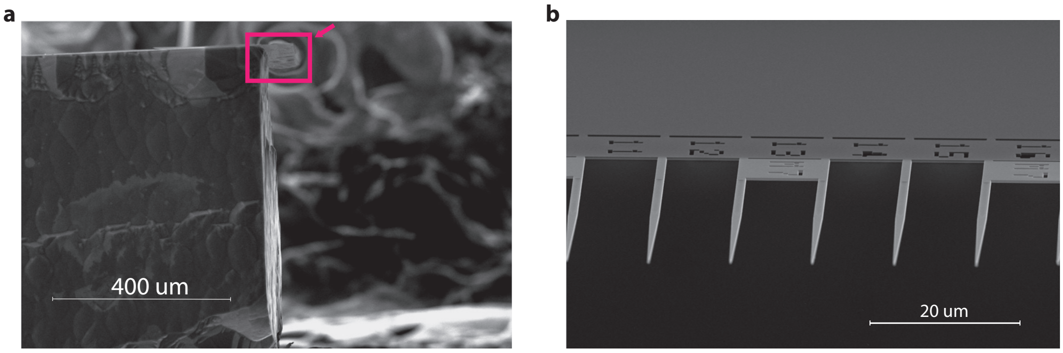

Fig. S1: **SEM image of the chip with nanophotonic devices. a**, The devices are marked with the red box and are overhanging by 80 μm at the edge of the 600 μm thick chip. The devices are undercut through the entire chip thickness by etching the chip from the side. **b,** A zoomed in SEM view from the direction of the arrow in **a**.

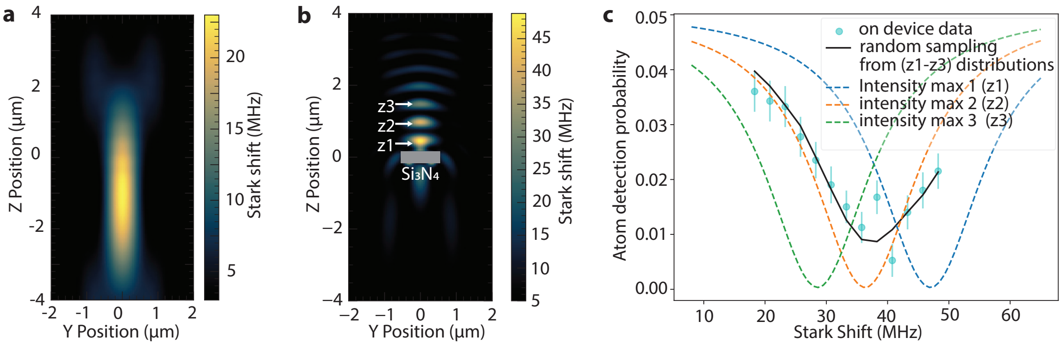

Fig. S2: **Stark shift.** **a**, Stark shift of the 895 nm D1 transition induced by a 2.4 mW, 935 nm optical tweezer with a waist of 1.1 μm. **b**, Modified trapping potential and the corresponding D1 Stark shift with the introduction of a silicon nitride device at the location marked. The tweezer is partially reflected by the device, resulting in the formation of a lattice-like potential with the closest intensity maximum (z1) causing the largest Stark shift. **c**, Expected Stark shift survival curves for the first three intensity maxima (z1-z3). The experimental curve is fitted to random sampling from the three survival-curves, with a probability of 0.29 to be in the first intensity maximum, 0.66 to be in the second intensity maximum, and 0.05 to be in the third intensity maximum.

of 330 nm, used in this work, the maximum loading efficiency to the first intensity maximum from Monte Carlo simulations is $\sim$40%. This loading efficiency is a function of the device thickness, atomic temperature, tweezer trap depth, angle of the devices with respect to the tweezer plane, and the adiabaticity of the tweezer movement while loading onto the devices. These further cause variations of loading probability into different pancakes across the array as seen in Fig. 3**b**. Further optimizations of these parameters can improve the loading efficiency. By performing Monte Carlo simulations of the Stark shifts experienced by the atoms in the various intensity maxima on top of the devices, we can estimate the proportion of atoms loaded into each of the intensity maxima. The dashed lines in Fig. S2**b** show the estimated Stark shifts experienced by atoms loaded into the three intensity maxima closest to the device surface (z1-z3). For the given atom, we fit our blowout curves probing the Stark shift on the devices assuming random sampling from these three curves to obtain the best-fit estimate of our loading distribution of 29% to the intensity maximum closest to the device, 66% to the second intensity maximum, and 5% to the third. The achievable detunings for the stark shift measurements are currently limited by AOM bandwidth.

2.1 Tweezer Objective Z Positioning and MOT Coil Stabilization

The process of loading atoms onto the nanophotonic devices from free space is sensitive to the focal plane of the tweezers relative to the plane of the devices. Therefore, we require control over the Z position of the objective. Towards this end, we have our objective on a translation stage and use a piezoelectric screw to actuate the stage forwards and backward to vary the focal plane of the tweezers.

In order to optimize the position of the objective, we block the tweezers with a sliding optics mount holding a 900 nm LED which we turn on to illuminate the nanophotonic chip through our tweezer objective and image it on our EMCCD. We then assign a focal score to the image. We calculate the focal score by first applying a bilateral filter to the image to reduce the noise and then calculating the Laplacian of the filtered image to give us a numeric value that is proportional to the contrast in the image which is maximized when the image is in focus.

In general, we do not see the best loading results at the exact image focus, so we scan through the focus to establish the maximum score and target a focus score relative to the measured maximum in order to counteract long timescale drift in the LED output power. We can also choose a subsection of the image to target different planes due to the small relative angle between the chip and the objective.

During the course of the experiment, the variation in the focal score is monitored to ensure the stability of the Z-position reference. The focal position originally shifted by a few microns over the course of a couple of hours. The source of this variation was found to be the temperature gradient generated by the MOT coils

during the experiment run time. To mitigate this problem, we stabilize the temperature around the coils and the objective region by maintaining a constant MOT on-off ratio during experiment cycles. When the experiment is not running the MOT coil currents are maintained to have the same effective heat output as running the experiment at the controlled duty cycle. With this careful temperature control in the vicinity of the objective and its translation stage, we achieve stability of the Z-position for up to 24 hours with less than 300 nm variation.

3 Atomic Lifetime and Imaging On Devices

To characterize the atomic lifetime in the tweezers, we load atoms in the tweezers and hold them for a variable amount of time before imaging. The corresponding probability to detect the atom as the tweezer hold time is varied is shown in Fig. S3**a**. From the fits, we estimate an atomic lifetime of 13.6 seconds in the loading region and 0.78 seconds when trapped in the standing wave traps on top of the devices. Reduction in the atomic lifetime in the standing wave traps on nanophotonic devices is consistent with the earlier observations of atoms trapped on top of nanophotonic devices [3], and is expected from thermally excited phononic modes of the devices [4]. This lifetime is however sufficiently large compared to observed coherence times on top of the devices of ∼2 ms [5], atom rearrangement times of ∼100 us, and the required atom-cavity interaction time for entangled photon generation ∼100 ns [6]. Before the installation of the chip inside the chamber, atomic lifetimes in excess of 60 seconds in free-space optical tweezers were observed, indicating a finite reduction in the lifetime with the chip placement and the associated increase in the atomic flux required to create the MOT in the presence of the chip. While imaging fidelities in excess of 99% are routinely obtained for atoms in the loading region, the imaging fidelity on the devices is reduced. An imaging histogram for an atom trapped on the device is shown in Fig. S3**b** demonstrating an imaging fidelity of 86%, approaching state of the art value of ∼95% obtained in [7]. While the background is comparable to that of atoms in the loading region, the atomic signal is shifted to lower photon numbers. This is due to an increased atomic loss during the imaging due to the modified trapping potential. This can be further optimized in future experiments by optimizing the tweezer power, imaging laser powers, and detunings. Additional methods such as employing Raman sideband cooling to achieve lower atomic temperatures or imaging with lower drive power for longer timescales as in [7] can improve this measurement.

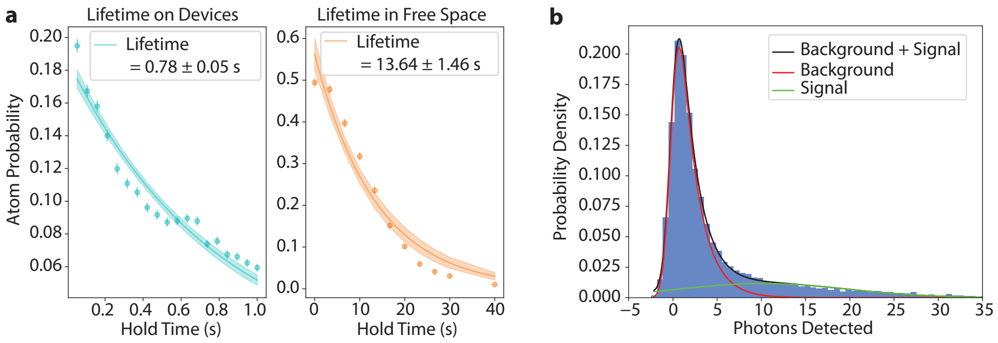

Fig. S3: **Atom lifetime and imaging on devices.** **a**, Atom survival in the tweezer as a function of holding time in the loading region (orange) and when trapped on top of the devices (cyan). Atoms are relatively long-lived even on top of the devices with lifetimes of individual atoms varying from 0.7 to 1 second. **b**, A typical single-atom imaging histogram while atoms are trapped on top of the devices. Here the fidelity of detection is estimated to be 86% with a threshold of 5 photons. Optimization of the tweezer power and imaging parameters can further improve the imaging fidelity.

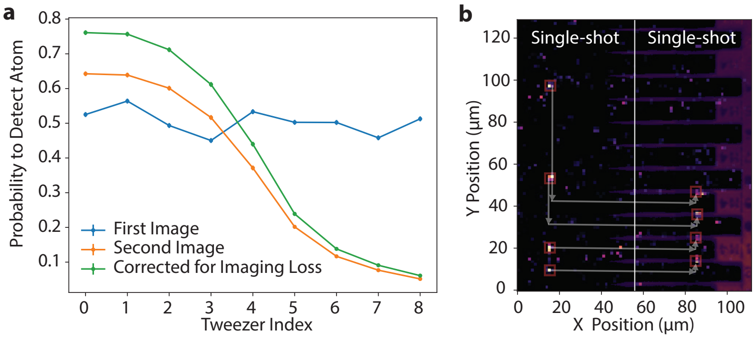

Fig. S4: **Rearrangement to the devices.** **a**, Rearrangement success in the compression part of the rearrangement to the devices. Stochastically loaded atoms (blue) are detected and compressed into a defect-free array (orange). Atom loss of 10-15% during the first image results in a finite reduction of the atom probability after rearrangement. The green line shows the rearrangement curve after correcting for the imaging losses. **b**, The atomic trajectory used for rearrangement in Fig. 4. Atoms are first compressed into a defect-free array, then moved in between devices, and finally moved on top of the devices.

4 Rearrangement

Loading atoms into the optical tweezer array is a stochastic process with a 55% loading probability. For deterministic placement of these atoms in a specific order onto a device or multiple devices, the stochastic loading pattern must be identified and moved to the target locations. We achieve this in a two-step process. In the first step, we identify the tweezers that were originally loaded with atoms and rearrange them into a defect-free configuration. In the second step, we move the defect-free array to the target location. In order to achieve a defect-free configuration of atoms, we first take an image of the stochastically loaded array to determine which tweezers have atoms and drop the tweezers that do not contain atoms. Following this, we chirp the remaining AOD frequencies that form our optical tweezers to compress the fully filled array into a defect-free configuration. We make this compression move in 1 ms following a symmetric piecewise quadratic frequency chirp profile. Blue points in Fig. S4**a** show the probability of stochastically loading each tweezer index. The orange points show the probability of detecting an atom at each tweezer site following the initial detection and rearrangement. A finite atomic loss during the first image and the losses due to the rearrangement result in non-unity atom probability after rearrangement. Around 10-15% of atoms are lost after the first round of imaging, resulting in a corresponding reduction of atomic probability in the second image. The green curve in Fig. S4**a** shows the probability to detect atoms at each site after rearrangement, correcting for the site-specific atomic loss during imaging. The remaining 23% atomic loss stems from the losses during rearrangement. The power imbalance between the tweezers during the process of rearrangement and specific frequency chirp profile can cause atoms to heat out of these traps during the first step. The imaging loss can be reduced by stroboscopic imaging and the rearrangement losses can be reduced by further characterization and optimization of the tweezer power, phase, chirp profiles, and trajectories enabling near unity rearrangement efficiencies.

After the first step of creating a defect-free array, atoms are then moved close to the target device using an X-direction move. This is followed by an adiabatic Y-direction move onto the devices. Fig. S4**b** shows the combined step 1 and step 2 trajectory of atoms. The left image shows the initial detection of the stochastically loaded atoms and their calculated trajectories. The right image shows the same atoms loaded on top of the device following the marked trajectories.

5 Free-space Coupling

The free-space coupling is achieved by designing the mode supported by the waveguide taper end to match that of a diffraction-limited spot created by a low numerical aperture lens as shown in Fig. S5**a**. Currently, a 40 mm aspheric lens placed outside the chamber is used to couple light in and out of the waveguides with a

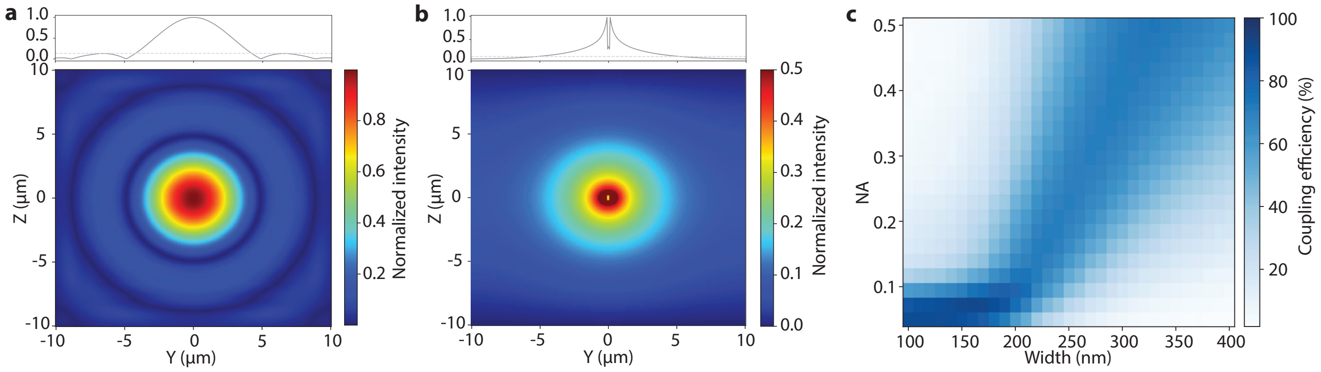

Fig. S5: **Free-space coupling.** **a**, Normalized 2D intensity profile of the diffraction-limited mode generated by a 0.17 NA lens. A line cut along the Y axis at Z=0 is shown above. The dashed horizontal line shows the $1/e^2$ amplitude. **b**, Second TE mode supported by the waveguide taper end. Corresponding line cut along the Y axis at Z=0 is shown above. The dashed horizontal line shows the $1/e^2$ amplitude. **c**, Estimated coupling efficiency from Lumerical FDTD simulation for a waveguide with a constant thickness of 330 nanometers.

Y-polarized input beam. This excites the second TE mode, shown in Fig. S5**b**, supported by the end of the taper. The linear, adiabatic taper then converts this mode into the mode supported by the cavity. Currently, we measure a coupling efficiency of 20%. Using a finite-difference time-domain (FDTD) calculation, we inject Gaussian beams generated by different numerical aperture (NA) lenses into waveguides of varying widths to estimate the power distribution across the waveguide modes. From this, we estimate that up to 91% coupling efficiency can be achieved using the free-space coupling technique. This requires optimal selection of the waveguide taper end width and numerical aperture of the coupling lens as shown in Fig. S5**c**. Coupling efficiencies up to 60% have been measured outside the chamber. However, limited optical access currently restricts the lenses and objectives that we can use for optimal coupling of the light beams. The external lens is mounted on a piezo-controlled stage allowing for coupling to any device of choice from outside the chamber. Fiber arrays or acousto-optical deflectors (AODs) [8] can be used to read out or probe multiple devices at the same time.

References

[1] Thompson, J. D. *et al.* Coupling a single trapped atom to a nanoscale optical cavity. *Science* **340**, 1202–1205 (2013).

[2] Luan, X. *et al.* The integration of photonic crystal waveguides with atom arrays in optical tweezers. *Advanced Quantum Technologies* **3**, 2000008 (2020).

[3] Samutpraphoot, P. *et al.* Strong coupling of two individually controlled atoms via a nanophotonic cavity. *Physical Review Letters* **124**, 063602 (2020).

[4] Hümmer, D., Schneeweiss, P., Rauschenbeutel, A. & Romero-Isart, O. Heating in nanophotonic traps for cold atoms. *Physical Review X* **9**, 041034 (2019).

[5] Dordević, T. *et al.* Entanglement transport and a nanophotonic interface for atoms in optical tweezers. *Science* **373**, 1511–1514 (2021).

[6] Menon, S. G., Singh, K., Borregaard, J. & Bernien, H. Nanophotonic quantum network node with neutral atoms and an integrated telecom interface. *New Journal of Physics* **22**, 73033 (2020).

[7] Meng, Y., Liedl, C., Pucher, S., Rauschenbeutel, A. & Schneeweiss, P. Imaging and localizing individual atoms interfaced with a nanophotonic waveguide. *Physical Review Letters* **125**, 053603 (2020).

[8] Pu, Y. *et al.* Experimental entanglement of 25 individually accessible atomic quantum interfaces. *Science advances* **4**, eaar3931 (2018).
